# 2-(Benzene­sulfonamido)pyridinium perchlorate

**DOI:** 10.1107/S1600536809019205

**Published:** 2009-05-29

**Authors:** Xun Li, Dan Xie, Zhi-Hong Xiao, Chang-Zhu Li

**Affiliations:** a School of Chemistry and Biological Engineering, Changsha University of Science & Technology, Changsha 410004, People’s Republic of China; b Hunan Provincial Research Center of Biodiesel Engineering and Technology, Changsha 410004, People’s Republic of China

## Abstract

In the title compound, C_11_H_11_N_2_O_2_S^+^·ClO_4_^−^, the dihedral angle between the benzene and pyridinium rings is 87.33 (10)°. An intra­molecular N—H⋯O inter­action, with an S=O-bonded O atom as receptor, occurs in the cation. In the crystal structure, ion pairs occur, being linked by strong N—H⋯O hydrogen bonds. The perchlorate anion plays a further role in the mol­ecular packing by accepting several weak C—H⋯O inter­actions.

## Related literature

For the synthesis, see: Li, Yang *et al.* (2008 ▶). For a related structure containing the same cation, see: Li & Li (2009 ▶). For related structures, see: Li *et al.* (2008*a* ▶,*b* ▶). For applications of pyridinium salts, see: Li *et al.* (2007 ▶); Li, Fan *et al.* (2008 ▶); Miyashita *et al.* (1977 ▶); Ganeshpure *et al.* (2007 ▶).
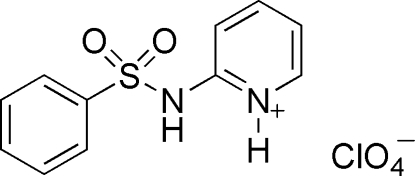


## Experimental

### 

#### Crystal data

C_11_H_11_N_2_O_2_S^+^·ClO_4_^−^
*M* *_r_* = 334.73
Triclinic, 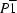

*a* = 5.6594 (11) Å
*b* = 7.5996 (15) Å
*c* = 16.157 (3) Å
α = 83.21 (3)°
β = 83.70 (3)°
γ = 73.84 (3)°
*V* = 660.6 (2) Å^3^
*Z* = 2
Mo *K*α radiation
μ = 0.48 mm^−1^
*T* = 113 K
0.20 × 0.10 × 0.08 mm

#### Data collection

Rigaku Saturn CCD diffractometer
Absorption correction: multi-scan (*CrystalClear*; Rigaku/MSC, 2005 ▶) *T*_min_ = 0.911, *T*_max_ = 0.963
5339 measured reflections
3051 independent reflections
2473 reflections with *I* > 2σ(*I*)
*R*_int_ = 0.024

#### Refinement

*R*[*F* ^2^ > 2σ(*F* ^2^)] = 0.034
*wR*(*F* ^2^) = 0.092
*S* = 1.07
3051 reflections
198 parameters
H atoms treated by a mixture of independent and constrained refinement
Δρ_max_ = 0.32 e Å^−3^
Δρ_min_ = −0.42 e Å^−3^

### 

Data collection: *CrystalClear* (Rigaku/MSC, 2005 ▶); cell refinement: *CrystalClear*; data reduction: *CrystalClear*; program(s) used to solve structure: *SHELXS97* (Sheldrick, 2008 ▶); program(s) used to refine structure: *SHELXL97* (Sheldrick, 2008 ▶); molecular graphics: *SHELXTL* (Sheldrick, 2008 ▶); software used to prepare material for publication: *CrystalStructure* (Rigaku/MSC, 2005 ▶).

## Supplementary Material

Crystal structure: contains datablocks global, I. DOI: 10.1107/S1600536809019205/hb2972sup1.cif

Structure factors: contains datablocks I. DOI: 10.1107/S1600536809019205/hb2972Isup2.hkl

Additional supplementary materials:  crystallographic information; 3D view; checkCIF report

## Figures and Tables

**Table 1:** Hydrogen-bond geometry (Å, °)

*D*—H⋯*A*	*D*—H	H⋯*A*	*D*⋯*A*	*D*—H⋯*A*
N1—H1*A*⋯O1	0.82 (2)	2.01 (2)	2.694 (2)	141 (2)
N2—H2*A*⋯O4	0.81 (2)	2.02 (2)	2.808 (2)	165 (2)
C1—H1⋯O3^i^	0.95	2.37	3.301 (3)	166
C3—H3⋯O5^ii^	0.95	2.44	3.303 (3)	151
C7—H7⋯O4^iii^	0.95	2.53	3.417 (2)	156
C8—H8⋯O3^iii^	0.95	2.58	3.251 (3)	128

Symmetry codes: (i) 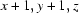; (ii) 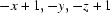; (iii) 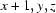.

**Fig. 1.**
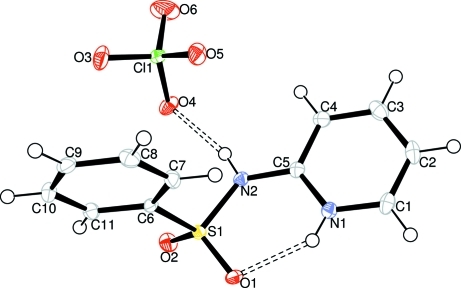
View of the molecular structure of (I) with displacement ellipsoids drawn at the 50% probability level and H atoms shown as small spheres of arbitrary radius. Dashed lines indicate hydrogen bonds.

## supplementary crystallographic information

### Comment

Organic pyridinium salts have been used as not only supramolecular guests (Li *et al.*, 2007; Li, Fan, Fan *et al.*, 2008), but also catalysts and/or media for esterification (Ganeshpure *et al.*2007; Miyashita *et al.* 1977). To seek a new pyridinium catalyst for biodiesel transformation, the title compound, (I), was synthesized and further determined by X-ray diffraction.

The title compound, (I), consists of a pyridinium cation and a perchlorate anion (Fig. 1). In the cation, the distance [1.383 (2) Å] between the pyridinum C atom and its connected amino N atom is slightly longer than the nitrate [1.378 (2) Å] (Li *et al.* 2009), also showing some conjugation of amino group with pyridinium ring. The benzene ring constructs an angle of 87.33 (10)° with the pyridinium ring, similar to the nitrate [87.59 (8)°]. The cation structure is stabilized by an intramolecular N—H···O hydrogen bond (Table 1), with S=O oxygen as H-bonding receptor, different from by C—H···O interaction in the nitrate.

Like the nitrate, in the crystal structure, a strong N—H···O hydrogen bond link the cation and anion (Table 1). The anion ClO_4_ plays a great important role in the molecular packing *via* weak C—H···O interactions (Table 1).

### Experimental

The title compound was prepared according to the reported literature (Li, Yang *et al.* 2008). Colourless needles of (I) were obtained by evaporation of a perchloric acid solution of the sulfonamide.

### Refinement

The H atoms bound to C were positioned geometrically (C—H = 0.95 Å)and refined as riding with *U*_iso_(H) = 1.2 *U*_eq_(C)]. The N—H hydrogen atoms were refined with their isotropic displacement parameters, and N—H distances are restrained to 0.82 (2) and 0.81 (2) Å, respectively.

### Crystal data

**Table:**

C_11_H_11_N_2_O_2_S^+^·ClO_4_^−^	*Z* = 2
*M**_r_* = 334.73	*F*(000) = 344
Triclinic, *P*1	*D*_x_ = 1.683 Mg m^−^^3^
Hall symbol: -P 1	Mo *K*α radiation, λ = 0.71073 Å
*a* = 5.6594 (11) Å	Cell parameters from 2172 reflections
*b* = 7.5996 (15) Å	θ = 2.8–27.9°
*c* = 16.157 (3) Å	µ = 0.48 mm^−^^1^
α = 83.21 (3)°	*T* = 113 K
β = 83.70 (3)°	Needle, colourless
γ = 73.84 (3)°	0.20 × 0.10 × 0.08 mm
*V* = 660.6 (2) Å^3^	

### Data collection

**Table:**

Rigaku Saturn CCD diffractometer	3051 independent reflections
Radiation source: rotating anode	2473 reflections with *I* > 2σ(*I*)
confocal	*R*_int_ = 0.024
Detector resolution: 7.31 pixels mm^-1^	θ_max_ = 27.9°, θ_min_ = 2.8°
ω and φ scans	*h* = −7→7
Absorption correction: multi-scan (*CrystalClear*; Rigaku/MSC, 2005)	*k* = −7→9
*T*_min_ = 0.911, *T*_max_ = 0.963	*l* = −19→21
5339 measured reflections	

### Refinement

**Table:**

Refinement on *F*^2^	Primary atom site location: structure-invariant direct methods
Least-squares matrix: full	Secondary atom site location: difference Fourier map
*R*[*F*^2^ > 2σ(*F*^2^)] = 0.034	Hydrogen site location: inferred from neighbouring sites
*wR*(*F*^2^) = 0.092	H atoms treated by a mixture of independent and constrained refinement
*S* = 1.07	*w* = 1/[σ^2^(*F*_o_^2^) + (0.0383*P*)^2^ + 0.3333*P*] where *P* = (*F*_o_^2^ + 2*F*_c_^2^)/3
3051 reflections	(Δ/σ)_max_ = 0.001
198 parameters	Δρ_max_ = 0.32 e Å^−^^3^
0 restraints	Δρ_min_ = −0.42 e Å^−^^3^

### Special details

**Table:**

Geometry. All e.s.d.'s (except the e.s.d. in the dihedral angle between two l.s. planes) are estimated using the full covariance matrix. The cell e.s.d.'s are taken into account individually in the estimation of e.s.d.'s in distances, angles and torsion angles; correlations between e.s.d.'s in cell parameters are only used when they are defined by crystal symmetry. An approximate (isotropic) treatment of cell e.s.d.'s is used for estimating e.s.d.'s involving l.s. planes.
Refinement. Refinement of *F*^2^ against ALL reflections. The weighted *R*-factor *wR* and goodness of fit *S* are based on *F*^2^, conventional *R*-factors *R* are based on *F*, with *F* set to zero for negative *F*^2^. The threshold expression of *F*^2^ > σ(*F*^2^) is used only for calculating *R*-factors(gt) *etc*. and is not relevant to the choice of reflections for refinement. *R*-factors based on *F*^2^ are statistically about twice as large as those based on *F*, and *R*- factors based on ALL data will be even larger.

### Fractional atomic coordinates and isotropic or equivalent isotropic displacement parameters (Å^2^)

**Table:**

	*x*	*y*	*z*	*U*_iso_*/*U*_eq_	
S1	0.22811 (8)	0.24398 (6)	0.16564 (3)	0.01285 (12)	
O1	0.3752 (3)	0.36817 (19)	0.13565 (8)	0.0188 (3)	
O2	−0.0260 (2)	0.2927 (2)	0.14890 (8)	0.0195 (3)	
N1	0.5339 (3)	0.3703 (2)	0.28668 (10)	0.0146 (3)	
N2	0.2351 (3)	0.2162 (2)	0.26820 (9)	0.0142 (3)	
C1	0.6774 (3)	0.4261 (3)	0.33434 (12)	0.0178 (4)	
H1	0.7775	0.5030	0.3096	0.021*	
C2	0.6782 (4)	0.3717 (3)	0.41768 (12)	0.0202 (4)	
H2	0.7815	0.4072	0.4513	0.024*	
C3	0.5244 (4)	0.2628 (3)	0.45284 (12)	0.0214 (4)	
H3	0.5208	0.2255	0.5111	0.026*	
C4	0.3781 (4)	0.2091 (3)	0.40365 (11)	0.0167 (4)	
H4	0.2729	0.1353	0.4276	0.020*	
C5	0.3861 (3)	0.2644 (3)	0.31822 (11)	0.0133 (4)	
C6	0.3713 (3)	0.0251 (2)	0.13242 (11)	0.0122 (3)	
C7	0.6069 (3)	−0.0679 (3)	0.15558 (11)	0.0167 (4)	
H7	0.6874	−0.0172	0.1915	0.020*	
C8	0.7216 (4)	−0.2369 (3)	0.12480 (11)	0.0185 (4)	
H8	0.8823	−0.3031	0.1397	0.022*	
C9	0.6018 (4)	−0.3089 (3)	0.07244 (12)	0.0179 (4)	
H9	0.6818	−0.4241	0.0514	0.021*	
C10	0.3665 (4)	−0.2150 (3)	0.05020 (12)	0.0193 (4)	
H10	0.2865	−0.2659	0.0142	0.023*	
C11	0.2487 (3)	−0.0472 (3)	0.08060 (11)	0.0157 (4)	
H11	0.0869	0.0175	0.0663	0.019*	
H1A	0.536 (4)	0.398 (3)	0.2358 (15)	0.022 (6)*	
H2A	0.152 (4)	0.150 (3)	0.2905 (14)	0.019 (6)*	
Cl1	0.06395 (8)	−0.20083 (6)	0.34300 (3)	0.01538 (12)	
O3	0.0680 (3)	−0.3209 (2)	0.28027 (10)	0.0267 (3)	
O4	−0.0763 (2)	−0.01507 (19)	0.31513 (9)	0.0209 (3)	
O5	0.3112 (3)	−0.1954 (2)	0.35352 (9)	0.0243 (3)	
O6	−0.0508 (3)	−0.2619 (3)	0.42029 (10)	0.0415 (5)	

### Atomic displacement parameters (Å^2^)

**Table:**

	*U*^11^	*U*^22^	*U*^33^	*U*^12^	*U*^13^	*U*^23^
S1	0.0155 (2)	0.0101 (2)	0.0123 (2)	−0.00192 (17)	−0.00278 (15)	−0.00055 (15)
O1	0.0296 (8)	0.0140 (7)	0.0144 (6)	−0.0102 (6)	−0.0003 (5)	0.0011 (5)
O2	0.0169 (7)	0.0168 (7)	0.0220 (7)	0.0020 (6)	−0.0067 (5)	−0.0018 (5)
N1	0.0152 (8)	0.0138 (8)	0.0152 (8)	−0.0041 (6)	−0.0009 (6)	−0.0024 (6)
N2	0.0156 (8)	0.0148 (8)	0.0139 (7)	−0.0075 (7)	0.0006 (6)	−0.0008 (6)
C1	0.0127 (9)	0.0145 (10)	0.0268 (10)	−0.0023 (7)	−0.0014 (7)	−0.0081 (8)
C2	0.0169 (9)	0.0203 (10)	0.0245 (10)	−0.0014 (8)	−0.0065 (7)	−0.0104 (8)
C3	0.0230 (10)	0.0221 (11)	0.0165 (9)	0.0006 (9)	−0.0036 (7)	−0.0052 (8)
C4	0.0190 (9)	0.0148 (10)	0.0152 (8)	−0.0032 (8)	0.0002 (7)	−0.0017 (7)
C5	0.0122 (8)	0.0097 (9)	0.0166 (8)	0.0003 (7)	−0.0012 (6)	−0.0029 (7)
C6	0.0149 (9)	0.0099 (9)	0.0119 (8)	−0.0035 (7)	−0.0007 (6)	−0.0004 (6)
C7	0.0171 (9)	0.0160 (10)	0.0173 (9)	−0.0032 (8)	−0.0056 (7)	−0.0020 (7)
C8	0.0179 (9)	0.0169 (10)	0.0170 (9)	0.0010 (8)	−0.0017 (7)	−0.0002 (7)
C9	0.0246 (10)	0.0108 (9)	0.0171 (9)	−0.0043 (8)	0.0017 (7)	−0.0009 (7)
C10	0.0241 (10)	0.0181 (10)	0.0187 (9)	−0.0088 (8)	−0.0029 (7)	−0.0049 (7)
C11	0.0152 (9)	0.0167 (10)	0.0159 (8)	−0.0051 (8)	−0.0032 (7)	−0.0005 (7)
Cl1	0.0175 (2)	0.0130 (2)	0.0159 (2)	−0.00511 (18)	−0.00268 (16)	0.00102 (16)
O3	0.0278 (8)	0.0179 (8)	0.0367 (8)	−0.0036 (7)	−0.0096 (6)	−0.0113 (6)
O4	0.0163 (7)	0.0104 (7)	0.0344 (8)	−0.0002 (6)	−0.0049 (6)	−0.0012 (6)
O5	0.0179 (7)	0.0235 (8)	0.0331 (8)	−0.0049 (6)	−0.0116 (6)	−0.0018 (6)
O6	0.0476 (11)	0.0515 (12)	0.0251 (8)	−0.0225 (10)	0.0047 (7)	0.0133 (8)

### Geometric parameters (Å, °)

**Table:**

S1—O2	1.4296 (14)	C4—H4	0.9500
S1—O1	1.4352 (15)	C6—C11	1.391 (3)
S1—N2	1.6488 (16)	C6—C7	1.393 (3)
S1—C6	1.7540 (19)	C7—C8	1.391 (3)
N1—C5	1.338 (2)	C7—H7	0.9500
N1—C1	1.356 (2)	C8—C9	1.384 (3)
N1—H1A	0.82 (2)	C8—H8	0.9500
N2—C5	1.383 (2)	C9—C10	1.388 (3)
N2—H2A	0.81 (2)	C9—H9	0.9500
C1—C2	1.361 (3)	C10—C11	1.384 (3)
C1—H1	0.9500	C10—H10	0.9500
C2—C3	1.395 (3)	C11—H11	0.9500
C2—H2	0.9500	Cl1—O6	1.4315 (16)
C3—C4	1.376 (3)	Cl1—O3	1.4358 (15)
C3—H3	0.9500	Cl1—O5	1.4403 (15)
C4—C5	1.394 (2)	Cl1—O4	1.4594 (15)
			
O2—S1—O1	119.45 (9)	N2—C5—C4	119.97 (17)
O2—S1—N2	106.71 (9)	C11—C6—C7	121.88 (18)
O1—S1—N2	106.24 (9)	C11—C6—S1	118.51 (14)
O2—S1—C6	108.52 (9)	C7—C6—S1	119.56 (14)
O1—S1—C6	110.07 (9)	C8—C7—C6	118.41 (18)
N2—S1—C6	104.81 (9)	C8—C7—H7	120.8
C5—N1—C1	122.75 (17)	C6—C7—H7	120.8
C5—N1—H1A	116.0 (17)	C9—C8—C7	120.05 (18)
C1—N1—H1A	121.3 (17)	C9—C8—H8	120.0
C5—N2—S1	129.79 (14)	C7—C8—H8	120.0
C5—N2—H2A	116.6 (16)	C8—C9—C10	120.91 (19)
S1—N2—H2A	112.8 (16)	C8—C9—H9	119.5
N1—C1—C2	119.98 (19)	C10—C9—H9	119.5
N1—C1—H1	120.0	C11—C10—C9	119.91 (18)
C2—C1—H1	120.0	C11—C10—H10	120.0
C1—C2—C3	118.80 (18)	C9—C10—H10	120.0
C1—C2—H2	120.6	C10—C11—C6	118.83 (18)
C3—C2—H2	120.6	C10—C11—H11	120.6
C4—C3—C2	120.39 (18)	C6—C11—H11	120.6
C4—C3—H3	119.8	O6—Cl1—O3	109.89 (11)
C2—C3—H3	119.8	O6—Cl1—O5	110.65 (10)
C3—C4—C5	119.16 (19)	O3—Cl1—O5	110.12 (10)
C3—C4—H4	120.4	O6—Cl1—O4	109.52 (11)
C5—C4—H4	120.4	O3—Cl1—O4	108.72 (9)
N1—C5—N2	121.08 (16)	O5—Cl1—O4	107.89 (9)
N1—C5—C4	118.89 (17)		
			
O2—S1—N2—C5	−141.28 (17)	O1—S1—C6—C11	−118.07 (15)
O1—S1—N2—C5	−12.81 (19)	N2—S1—C6—C11	128.06 (15)
C6—S1—N2—C5	103.73 (18)	O2—S1—C6—C7	−168.22 (15)
C5—N1—C1—C2	1.1 (3)	O1—S1—C6—C7	59.37 (17)
N1—C1—C2—C3	−1.7 (3)	N2—S1—C6—C7	−54.50 (17)
C1—C2—C3—C4	1.1 (3)	C11—C6—C7—C8	0.7 (3)
C2—C3—C4—C5	0.2 (3)	S1—C6—C7—C8	−176.66 (14)
C1—N1—C5—N2	177.48 (17)	C6—C7—C8—C9	0.0 (3)
C1—N1—C5—C4	0.2 (3)	C7—C8—C9—C10	−0.3 (3)
S1—N2—C5—N1	9.9 (3)	C8—C9—C10—C11	−0.1 (3)
S1—N2—C5—C4	−172.85 (14)	C9—C10—C11—C6	0.8 (3)
C3—C4—C5—N1	−0.8 (3)	C7—C6—C11—C10	−1.1 (3)
C3—C4—C5—N2	−178.17 (17)	S1—C6—C11—C10	176.28 (14)
O2—S1—C6—C11	14.34 (17)		

### Hydrogen-bond geometry (Å, °)

**Table:**

*D*—H···*A*	*D*—H	H···*A*	*D*···*A*	*D*—H···*A*
N1—H1A···O1	0.82 (2)	2.01 (2)	2.694 (2)	141 (2)
N2—H2A···O4	0.81 (2)	2.02 (2)	2.808 (2)	165 (2)
C1—H1···O3^i^	0.95	2.37	3.301 (3)	166
C3—H3···O5^ii^	0.95	2.44	3.303 (3)	151
C7—H7···O4^iii^	0.95	2.53	3.417 (2)	156
C8—H8···O3^iii^	0.95	2.58	3.251 (3)	128

Symmetry codes: (i) *x*+1, *y*+1, *z*; (ii) −*x*+1, −*y*, −*z*+1; (iii) *x*+1, *y*, *z*.
